# Negative self-appraisal mediates the relationship between mindfulness and confidence among adolescent female provincial hockey players in South Africa

**DOI:** 10.17159/2078-516X/2019/v31i1a4371

**Published:** 2019-01-01

**Authors:** S Walker

**Affiliations:** Unit for Professional Training and Services in the Behavioural Sciences (UNIBS), University of the Free State, Bloemfontein, South Africa

**Keywords:** dispositional mindfulness, mediation

## Abstract

**Background:**

Mounting evidence suggests that mindfulness is positively related to athletic performance and athlete wellbeing. However, few attempts have been made to explore the psychological processes by which mindfulness might impact performance.

**Objective:**

To determine whether negative self-appraisal mediates the relationship between dispositional mindfulness and the confidence component of mental toughness among provincial adolescent female hockey players in South Africa.

**Methods:**

Provincial adolescent female hockey players (n=486) completed measures of dispositional mindfulness, mental toughness-related confidence and negative self-appraisal. Correlation coefficients were calculated between all variables included in the study. An ordinary least-squares regression analysis was performed to test the indirect effect of negative self-appraisal on the relationship between dispositional mindfulness and confidence.

**Results:**

Negative self-appraisal exhibited an indirect effect on the relationship between dispositional mindfulness and the confidence component of mental toughness (***β*** = .06, *SE* = .0, *CI*_95_ = .04, .09). A subsequent Soble test confirmed that negative self-appraisal served as a statistically significant mediator (***β*** = .06, *SE* = .01, *Z* = 5.76, *p* = .001) in the model. Furthermore, 78.3% of the variance in the effect of dispositional mindfulness on the confidence component of mental toughness was accounted for by negative self-appraisal.

**Conclusion:**

The effect of dispositional mindfulness on the confidence component of mental toughness among adolescent athletes is mediated by negative self-appraisal. Based on the current findings, dispositional mindfulness may foster confidence by lessening the impact of rigid negative appraisals of one’s performance and worth as an athlete.

Mindfulness is perhaps most often thought of as awareness of the present moment characterised by an accepting and non-judgemental orientation towards external stimuli and internal experiences.^[1]^ Dispositional mindfulness (DM) has been defined as an innate and relatively stable focus and quality of attention that is distinct from other forms of mindfulness and is associated with, yet conceptually unique from, various personality traits.^[2]^ DM has been associated with improved or superior performance in a number of sports.^[3]^ DM also appears to promote emotional resilience and well-being among athletes. For example, a negative association has been found between sport-related burnout and DM among competitive youth tennis players.^[4]^ Despite this growing body of literature on DM in sport and performance psychology, little is known about its mechanisms of impact. It has been theorised that mindfulness facilitates improved athletic performance by way of attentional awareness, experiential acceptance, cognitive flexibility and emotional regulation.^[5–6]^ Athletes with higher levels of DM are hypothesised to be more proficient at adopting a non-judgemental stance toward their inner experiences and physical performance. In addition, DM is thought to facilitate improved performance by promoting adaptive and flexible patterns of thinking.^[5]^ A recent study demonstrated that athletes who reported higher levels of DM were better at regulating their emotions and less inclined to engage in unproductive, repetitive thought.^[6]^ This, in turn, was associated with improved sport-specific coping. Based on the above discussion, further exploration of the mechanisms by which DM may impact upon specific performance-related psychological constructs appears warranted.

The research on DM and specific performance-related constructs such as mental toughness, (MT) is in its infancy. However, DM has been positively associated with MT in female adolescent hockey players.^[7]^ Notwithstanding conceptual debates regarding the precise nature of the construct, a number of theorists regard confidence or self-efficacy as an integral component of MT.^[8–9]^ In addition, confidence has long been considered critical to success in competitive athletics.^[10]^ Confidence is viewed as being central to not only the precise execution of sport-specific skills, but to the development of fundamental movement skill proficiency among adolescents.^[11]^ Given the apparent importance of confidence in the acquisition of basic athletic competencies and the execution of advanced skills, it seems logical to explore the contribution that mindfulness might make to confidence. Moreover, the importance that athletes and coaches place on confidence suggests that interventions directly linked to this particular construct would be more readily accepted and applied in competitive sports settings.

Within the context of MT, confidence is viewed as an athlete’s belief in their ability to effectively cope with and overcome challenges in their sport.^[9]^ This conceptualisation of confidence seems to be primarily informed by theories on cognitive appraisal and coping.^[12–13]^ Confidence could thus be viewed as an individual’s subjective appraisal of their ability to successfully meet a specific challenge. Such appraisals are influenced by beliefs that individuals hold about themselves, others and the world. Individual’s appraisals of their ability to deal with challenges are most functional when they are based on the accurate interpretation of information in a specific situation.^[12]^ Less confidence-consistent beliefs tend to be rigid and absolutistic. A tendency to view oneself as defined in totality by successes and failures stems from rigid self-appraisal beliefs which are inconsistent with developing a sense of confidence in one’s abilities.^[13]^

Adolescents are generally considered to be at increased risk of low self-confidence, partially as a result of an increased susceptibility to overly rigid and critical self-appraisal.^[13–14]^. It has also been suggested that female adolescent athletes frequently find themselves in contexts which might influence or promote negative self-appraisal.^[15]^ Female adolescents thus seem to be at an increased risk of engaging in rigid and overly critical self-appraisal. This, in turn, could be hypothesised to lead to a decrease in certain components of MT when related in this athlete population. Consequently, the association between DM and MT demonstrated in certain adolescent female athlete populations might be effected via self-appraisal beliefs.^[7]^ In other words, negative self-appraisal may mediate the relationship between DM and the confidence component of MT in this population. The current study aims to investigate this hypothesis in a sample of adolescent female provincial hockey players.

## Methods

### Participants

The relevant institutional body granted ethical clearance for the study. The South African Hockey Association granted permission for data to be collected at annual female under-16 and under-19 interprovincial tournaments. Informed consent was obtained from all participants, as well as from the guardians of all minors, prior to data collection. Participants completed the measures listed below between games or in the evenings.

Four hundred and eighty-six adolescent female provincial hockey players consented to participate in the study. The average age of the participants was 16.2 years (SD **±** 2.5). Participants reported having competed in an average of 2.4 interprovincial tournaments (SD **±** 1.4). Six percent of the sample had previously been selected for a South African hockey team at age group level.

### Measures

DM was measured using the Child and Adolescent Mindfulness Measure (CAMM).^[16]^ This 10-item self-report inventory requires respondents to endorse response options along a five-point Likert-type scale anchored by “never true” and “always true”. The CAMM yields a unitary mindfulness score, with higher scores indicative of higher levels of DM. Acceptable internal reliability has been reported for the CAMM in this population.^[7]^

To date, no measure of MT appears to have been developed specifically for use amongst adolescents or to have been normed in this population. Consequently, the current study employed the confidence scale of the Sport Mental Toughness Questionnaire (SMTQ) in order to measure this component of MT.^[8]^ Response options ranging from “not at all true” to “very true” are presented along a four-point Likert-type scale. The scale is scored by summing responses across the six items. Higher scores indicate increased confidence. Despite being developed for use among adults, the confidence scale of the SMTQ has demonstrated acceptable internal consistency in female adolescent athletes.^[7]^

The Self-downing factor identified on the revised version of the Child and Adolescent Scale of Irrationality (CASI-R) was employed as a measure negative self-appraisal.^[17]^ This factor is composed of eight items. Response options are presented along a five-point Likert-type scale anchored by “strongly disagree” and “strongly agree”. The CASI-R is scored by reversing the rationally worded items and then summing scores across all items. Higher scores are indicative of higher levels of irrational and absolutist thinking. The Self-downing factor of the CASI-R has demonstrated acceptable reliability in a non-clinical adolescent sample.^[17]^

### Statistical analysis

**Fig. 1:**
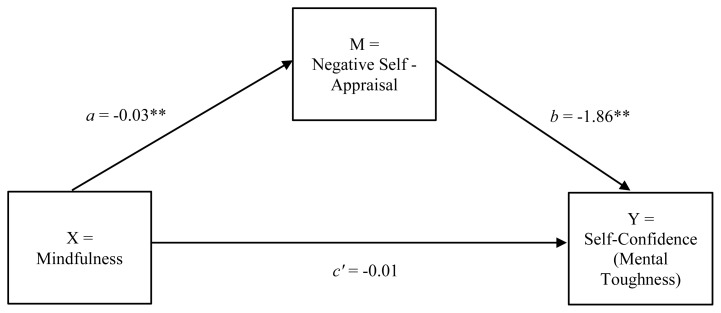
Negative self-appraisal mediates the relationship between mindfulness and the self-confidence component of mental toughness. ** p **<**0.01

Initially, internal reliability coefficients were calculated for all variables included in the study. Next, correlations between the CAMM total score, the CASI-R Self-downing factor and the SMTQ confidence scale were calculated. Finally, it was hypothesised that negative self-appraisal would mediate (indirect effect) the direct effect of DM on the confidence component of MT (see Figure 1). Consequently, a mediation analysis was conducted to test for the indirect effect of negative self-appraisal on the relationship between DM and the confidence component of MT. A path-analytic approach using Ordinary Least–Squares (OLS) regression analysis was employed for this purpose.^[18]^ The OLS regression analysis was performed using the PROCESS software macro for SPSS.^[18]^ The statistical significance of the cross-product of coefficients was tested using a nonparametric bootstrapping method. Consequently, no assumptions needed to be made with regard to the distribution of scores in the sample. Bias-corrected bootstrap procedures utilising 50 000 simulations were computed for the model. The significance of the indirect effect was determined using a 95% confidence interval.

## Results

Correlations between the SMTQ confidence scale, CAMM and CASI-R Self-downing factor are displayed in Table 1. Mean scores, standard deviations (SDs) and internal consistency coefficients for each of the measures are also reported. It is apparent from Table 1 that all internal consistency coefficients meet the prescribed minimum level of acceptability for non-cognitive measures (**α ≥** .70).^[19]^ A statistically significant (*p* **≤** .05) positive correlation was found between DM and the confidence component of MT. Conversely, negative self-appraisal was significantly (*p* **≤** .01) and negatively correlated with both DM and the confidence component of MT.

Given that the three variables of interest were significantly correlated, the proposed mediation model (see Fig. 1) could be tested. Model coefficients for the mediation analysis are presented in Table 2. DM demonstrated a significant negative association with negative self-appraisal (path *a*) and negative self-appraisal demonstrated a significant negative association with the confidence component of MT (path *b*) (see Fig. 1). The direct effect (path *c’*) of DM on the confidence component of MT was no longer significant when negative self-appraisal was accounted for. Results based on the bias-corrected bootstrap method indicate the indirect effect of negative self-appraisal on the relationship between DM and the confidence component of MT (***β*** = .06, *SE* = .01, *CI_95_* = .04, .09). In addition, the test of the indirect effect based on the Sobel test confirms that negative self-appraisal is a statistically significant mediator (***β*** = .06, *SE* = .01, *Z* = 5.76, *p* = .001) in this model. A total of 78.3% of the variance in the effect of DM on the confidence component of MT was accounted for by negative self-appraisal.

## Discussion

An established body of clinical and sports psychology literature implicates rigid and generalised ways of thinking in poor self-confidence and reduced sporting performance.^[9–10, 14]^ Consequently, the current finding that negative self-appraisal demonstrated a significant negative relationship with the confidence component of MT is not surprising. Results of the initial correlational analyses also highlighted the positive relationship between DM and the confidence component of MT. This finding is consistent with existing research on the relationship between DM and desirable performance-related psychological processes and states.^[3,6–7]^ More specifically, these findings suggest that confidence may be one mechanism by which DM may impact athletic performance. Given the value placed upon confidence by both athletes and coaches, mindfulness-based sport psychology interventions appear to be increasingly deserving of exploration.

DM was also found to exhibit a significant inverse relationship with negative self-appraisal. Increased levels of DM were associated with a tendency for participants to be less self-critical, less judgemental and less cognitively rigid. This would seem to support the prevailing notion that mindfulness functions to reduce the impact of absolutistic and categorical thinking.^[5–6]^ Mindfulness interventions might thus prove a credible alternative to cognitive interventions aimed at reducing the impact of rigid and absolutistic thinking on athletic performance and well-being. At the very least, mindfulness interventions may prove useful adjuncts to cognitive-behavioural sport psychology interventions. For example, DM might foster athletes’ awareness of their tendency towards rigid self-appraisal, as well as tracking the moment-to-moment effect that engaging with these appraisals has on performance.^[3,6]^

The findings discussed above generally reaffirm existing knowledge. The unique contribution of the current study is the exploration of negative self-appraisal as a possible mechanism by which DM might impact the confidence component of MT. Of particular interest was the possibility that self-appraisal functioned as a potential cognitive process through which DM impacted confidence, specifically within the context of MT. This was shown to be the case among adolescent female provincial hockey players. Self-appraisal mediated the effect of DM on confidence. More specifically, an increase in DM results in a reduction in negative self-appraisal which in turn has a positive effect on confidence. Causal conclusions should not be drawn from cross-sectional data. However, it appears that one way in which DM positively impacts confidence among female athletes is by decreasing the frequency and/or intensity of negative self-appraisals. This interpretation would be consistent with theoretical accounts of how DM might impact various aspects of athletic performance.^[5–6]^ Current opinion would seem to suggest that mindfulness would not necessarily result in a change in the content or believability of negative self-appraisals that athletes might hold or experience. However, higher DM might be expected to decrease the extent to which athletes engaged with or became attached to any form of self-appraisal.^[1,3,6]^ This implies that DM would be expected to facilitate a more accepting attitude towards rigid negative and positive self-appraisals of athletes’ performance and ability. Consequently, DM should not be viewed as a mechanism by which to replace negative self-appraisal with positive self-appraisal, but rather as a means of negating the tendency to cognitively and emotionally over-engage with such self-appraisals.

It is important to note the magnitude of the mediation effect that negative-self appraisal has on the relationship between DM and the confidence component of MT. Negative self-appraisal accounts for 78% of the variance of the effect of DM on confidence. Consequently, in the model that was tested in this study, DM seems to impact the confidence component of MT primarily via negative self-appraisal. This would suggest that any variability in confidence brought about by changes in DM amongst female adolescent hockey players is predominantly the result of changes in the frequency and/or intensity of rigid and absolutistic negative self-appraisal. Stated differently, DM seems to influence MT-related confidence predominantly via the intensity, frequency, believability and/or attachment to negative-self appraisal. The utility of mindfulness-based interventions aimed at increasing confidence among adolescent female athletes seems to be justifiable from both mindfulness and traditional cognitive-behavioural paradigms.^[5–6, 14]^ Moreover, mindfulness-based interventions might hold promise in non-performance-related areas of female athlete health. Many issues relating to body image and eating behaviour have been shown to be largely maintained by pervasive patterns of negative self-evaluation.^[15]^ Consequently, if mindfulness interventions are able to impact upon negative self-appraisal, it seems logical that such interventions might prove effective in addressing some of the most disabling mental health issues facing adolescent female athletes.

### Study limitations

The current study highlights the potential for DM to mediate the impact that rigid, absolutistic self-appraisals have on MT-related confidence. However, these findings cannot be generalised beyond adolescent female athletes. Nor should these findings be considered generalizable to more individual sporting contexts. Further research is needed to determine whether negative self-appraisal mediates the relationship between DM and confidence in other athlete populations and in contexts other than team sports. In addition, the current study investigated the relationship between DM, negative self-appraisal and MT-related confidence. Single case series and controlled intervention studies would go a long way towards demonstrating whether mindfulness-based interventions lead to improved confidence by reducing the intensity, frequency and believability of rigid and global negative self-appraisal.

The findings of the current study should only be interpreted within the constraints of the specific model that was tested. Consequently, no inferences can be drawn regarding the possible mediating role of constructs other than negative self-appraisal. Similarly, the utility of constructs other than DM to influence confidence via negative self-appraisal have not been determined. Studies that explore the conditional interactions between multiple psychological processes and changes in confidence are required. The impact of DM on metacognitive processes, such as intolerance of uncertainty and rumination, as well as their subsequent impact on confidence, would be a fruitful avenue of future research.

Finally, while data were collected via developmentally appropriate measures of DM and self-appraisal, the confidence component of MT was measured with an instrument intended for use with adult populations. Although little research has been conducted in this area, it is plausible that developmentally-related differences in MT exist between adolescents and adults. Consequently, these findings cannot be considered to be the final word on the interaction between DM, self-appraisal and MT in adolescents.

## Conclusion

The current study furthers the understanding of mechanisms through which DM impacts upon athletic performance. DM has been demonstrated to result in lower levels of negative self-appraisal which, in turn, result in an increase in adolescent female athletes' confidence to meet the challenges of competitive sport. The promotion of mindfulness thus appears to provide an avenue through which to lessen the impact of patterns of rigid thinking on the confidence of adolescent female athletes.

## Figures and Tables

**Table 1:** Correlations, descriptive statistics and reliability coefficients for the study variables (n=486)

Variables	SMTQ confidence	CASI-R SD	CAMM Total
SMTQ confidence	-	−0.39**	0.11*
CASI-R SD		-	−0.324**
**α**	0.72	0.81	0.74
M	17.60	18.00	22.19
SD	3.02	5.84	6.05

** p**<** 0.01;

* p**<** 0.05

*M, Mean; SD, Standard deviation; SMTQ confidence, Sport Mental Toughness Questionnaire Confidence Score; CASI-R SD, Revised Child and Adolescent Scale of Irrationality Self-downing factor; CAMM, Child and Adolescent Mindfulness Measure*

**Table 2:** Model coefficients for the mediation analysis (n=486)

	Consequence													
	M (Negative self-appraisal)							Y (Confidence component of MT)						
Antecedent		Coeff.	SE	p	F	dfs	R2		Coeff.	SE	p	F	dfs	R2
X (Mindfulness)	a	−0.03	0.00	**0.001<**	56.67	1; 484	0.10	c’	−0.01	0.02	0.70	-	-	-
M (Negative self-appraisal)		-	-	-	-	-	-	b	−1.86	0.21	**0.001<**	44.16	2; 483	0.15
Constant	i1	2.77	0.11	**0.001<**	-	-		i2	21.50	0.75	**0.001<**	-	-	-

F, F-value; Coeff, Coefficient

## References

[1] Bishop SR Lau M Shapiro S Mindfulness: A proposed operational definition Clin Psychol Sci Pract 2004 11 3 230 241 10.1093/clipsy.bph077

[2] Rau HK Williams PG Dispositional mindfulness: A critical review of construct validation research Pers Individ Dif 2016 93 1 32 43 10.1016/j.paid.2015.09.035

[3] Sappington R Longshore K Systematically reviewing the efficacy of mindfulness-based interventions for enhanced athletic performance J Clin Sport Psychol 2015 9 3 232 262 10.1123/jcsp.2014-0017

[4] Walker SP Mindfulness and burnout among competitive adolescent tennis players S Afr J Sports Med 2013 25 4 105 108 10.7196/SAJSM.498

[5] Birrer D Röthlin P Morgan G Mindfulness to enhance athletic performance: theoretical considerations and possible impact mechanisms Mindfulness 2012 3 3 235 10.1007/s12671-012-0109-2

[6] Josefsson T Ivarsson A Lindwall M Mindfulness mechanisms in sport: Mediating effects of rumination and emotion regulation on sport-specific coping Mindfulness 2017 8 5 1354 1363 10.1007/s12671-017-0711-4 28989551 PMC5605575

[7] Walker SP Mindfulness and mental toughness among provincial adolescent female hockey players S Afr J Sports Med 2016 28 2 46 50 10.17159/2078-516X/2016/v28i2a1110

[8] Sheard M Golby J van Wersch A Progress toward construct validation of the Sport Mental Toughness Questionnaire (SMTQ) Euro J Psychol Assess 2009 25 3 186 193 10.1027/1015-5759.25.3.186

[9] Clough PJ Earle K Sewell D Mental toughness: the concept and its measurement Cockerill I Solutions in sport psychology London Thomson 2002 32 45

[10] Vealey RS Chase MA Self-confidence in sport: Conceptual and research advances 3rd ed Horn TS Advances in sport psychology Champaign, IL Human Kinetics 2008 65 97

[11] McGrane B Belton S Powell D The relationship between fundamental movement skill proficiency and physical self-confidence among adolescents J Sports Sci 2017 35 17 1709 1714 10.1080/02640414.2016.1235280 28282760

[12] Lazarus RS Folkman S Stress, appraisal, and coping New York Springer Pub. Co 1984

[13] DiGiuseppe RA Doyle KA Dryden W A practitioner’s guide to rational emotive behavior therapy 3rd ed New York Oxford University Press 2013

[14] Turner MJ Barker JB Using rational emotive behavior therapy with athletes Sport Psychol 2014 28 1 75 90 10.1123/tsp.2013-0012 28581692

[15] Arthur-Cameselle J Sossin K Quatromoni P A qualitative analysis of factors related to eating disorder onset in female collegiate athletes and non-athletes Eat Disord 2017 25 3 199 215 10.1080/10640266.2016.1258940 27897463

[16] Greco LA Baer RA Smith GT Assessing mindfulness in children and adolescents: development and validation of the Child and Adolescent Mindfulness Measure (CAMM) Psychol Assess 2011 23 3 606 614 10.1037/a0022819 21480722

[17] Bernard ME Cronan F The Child and Adolescent Scale of Irrationality: Validation data and mental health correlates J Cognit Psychother 1999 13 2 121 132 10.1891/0889-8391.13.2.121

[18] Hayes AF Introduction to mediation, moderation, and conditional process analysis: A regression-based approach New York Guilford Press 2013

[19] Foster J Parker I Carrying out investigations in psychology: methods and statistics Leicester Wiley-Blackwell 1995

